# Intraocular Pressure Reduction Is Associated with Reduced Venous Pulsation Pressure

**DOI:** 10.1371/journal.pone.0147915

**Published:** 2016-01-29

**Authors:** William H. Morgan, Philip H. House, Martin L. Hazelton, Brigid D. Betz-Stablein, Balwantray C. Chauhan, Ananth Viswanathan, Dao-Yi Yu

**Affiliations:** 1 Lions Eye Institute, University of Western Australia, Nedlands, Australia; 2 Statistics and Bioinformatics Group, Institute of Fundamental Sciences, Massey University, Palmerston North, New Zealand; 3 Department of Ophthalmology and Visual Sciences, Dalhousie University, Halifax, Nova Scotia, Canada; 4 Moorfields Eye Hospital, City University, London, United Kingdom; Bascom Palmer Eye Institute, University of Miami School of Medicine;, UNITED STATES

## Abstract

**Purpose:**

To explore whether alterations in intraocular pressure (IOP) affect vein pulsation properties using ophthalmodynamometric measures of vein pulsation pressure.

**Patients and Methods:**

Glaucoma patients had two retinal vein pulsation pressure (VPP) measurements from upper and lower hemiveins performed by ophthalmodynamometry at least 3 months apart. All subjects had VPP and IOP recorded at two visits, with standard automated perimetry, central corneal thickness (CCT) recorded at the initial visit. Where venous pulsation was spontaneous ophthalmodynamometry could not be performed and VPP was considered equal to IOP. Change in VPP was calculated and binarized with reduction in pressure scored 1 and no change or increase scored as 0. Data analysis used a mixed logistic regression model with change in VPP as response variable and change in IOP, visual field loss (mean deviation), CCT and time interval as explanatory variables.

**Results:**

31 subjects (20 females) with mean age 60 years (sd 11) were examined with change in VPP being significantly associated with change in IOP (odds ratio 1.6/mmHg, 95% CI 1.2 to 2.1 in the glaucoma patients but not suspect patients (p = 0.0005).

**Conclusion:**

Change in VPP is strongly associated with change in IOP such that a reduced intraocular pressure is associated with a subsequent reduction in VPP. This indicates that reduced IOP alters some retinal vein properties however the nature and time course of these changes is not known.

## Introduction

Glaucoma is a common blinding disease with current monitoring relying upon the detection of permanent loss of tissue or function.[1] Changes in retinal venous pulsation properties are associated with glaucoma severity and progression, and provide a possible quantitative method for estimating treatment adequacy and disease stability.**[2, 3]** It remains unknown whether these venous changes are fixed in disease or can be altered by therapy. Intraocular pressure (IOP) reduction is known to be beneficial in glaucoma therapy but it is not known what effect this has upon retinal venous properties.

Spontaneous retinal venous pulsation is absent in 46% of glaucoma subjects but only 2 to 10% of normal subjects, as revealed by several recent studies.[2, 4, 5] In such cases the veins can generally be induced to pulsate by increasing the intraocular pressure. The minimum additional pressure required to induce venous pulsation can be measured with an ophthalmodynamometer using the known calibration constant (0.32mmHg/g) to convert the applied force (g) into ophthalmodynamometric pressure (ODP).[6] The induced IOP at which venous pulsation occurs, known as vein pulsation pressure (VPP), is calculated by adding ODP to baseline IOP.[6] The VPP is associated with glaucoma severity and future glaucoma progression.[3]

The interaction between venous pulsation properties and glaucoma is not clearly understood. Modelling experiments suggest that elevated cerebrospinal fluid pressure or retinal venous resistance are the likely causes of absent venous pulsation and raised VPP.[7] Cerebrospinal fluid pressure is reduced in some patients with glaucoma and is an unlikely cause of this phenomenon.[8] Both extrinsic venous compression due to surrounding tissue swelling and intrinsic venous occlusion are known to reduce or eliminate spontaneous venous pulsation and cause an elevated VPP.[9, 10] In glaucoma, extrinsic causes are possible with some laminar volume increase seen early and lamina distortion seen later in the disease.[11]^,^[12] Intrinsic causes with elevated shear stress induced endothelial changes leading to vessel wall narrowing are also possible leading to an increased risk of venous occlusion.[9, 13–15] Elevated venous pulsation pressure may also reduce ocular perfusion pressure [16]. More recently, we have shown that the change in VPP alters the prognosis such that a reduction in VPP tends to reduce the risk of progression.[17] We wished to explore whether therapeutic IOP reduction results in a change in VPP and determine whether VPP may be a useful marker of treatment effect.

## Materials and Methods

All patients gave informed consent under the auspices of the University of Western Australia Human Ethics Committee with all data being kept on a secure computer database. The University of Western Australia Human Ethics Committee specifically approved this study and the consent procedure. Participants provided written informed consent to participate in this study. Each participant was given a detailed document outlining the study and procedure, which they read, could question and then signed. We developed a new ophthalmodynamometer, which has been described and calibrated in a recently published calibration trial in which glaucoma patients were invited to participate.[6] At both the invitation and calibration visits, IOP was measured and at the calibration trial visit ophthalmodynamometry was performed along with central corneal thickness and visual field testing. Those subjects were given usual care with standard follow up at 3 to 4 month intervals. Subsequently we began enrolling subjects in a five-year Vein Pulsation Study Trial in Glaucoma (VPSG) and many calibration trial subjects were invited to participate. All subjects were required to have primary open angle glaucoma (including pseudoexfoliation) in at least one eye determined by; 1: visual field change matching optic disk rim thinning or excavation; 2: glaucomatous change in optic disk from baseline using confocal scanning laser tomography or stereo-disk photography; 3: or both. One exception was that patients with 2 or more recorded optic disk haemorrhages with suspicious disk features were also included. Subjects must have had mild to moderate glaucoma (mean deviation required to be better than -18dB) in both eyes, open angles, no other ocular disorders, clear ocular media and visual acuity 20/40 or better. All subjects had either glaucoma, as defined above, or glaucoma suspect status in the fellow eye. Glaucoma suspect was defined as either a recorded IOP greater than 21 on 2 or more occasions or had suspicious optic disk features of rim notching and or excavation.

The calibration trial visit was defined as the initial visit and was separate to the primary invitation visit when IOP was measured. The VPSG enrolment visit was defined as the final visit for the purpose of this study and was separate to the secondary invitation visit when IOP was measured. The invitation visits occurred within 4 weeks of initial and final visits. The patient’s data were included in the study if more than 3 months had elapsed between the initial and final visits and if standard automated perimetry had been performed within three months of the first visit. The standard automated perimetry test was Humphrey SITA standard 24–2 visual field test, which was a second or subsequent field test in that patient’s experience.

**Fig 1:**
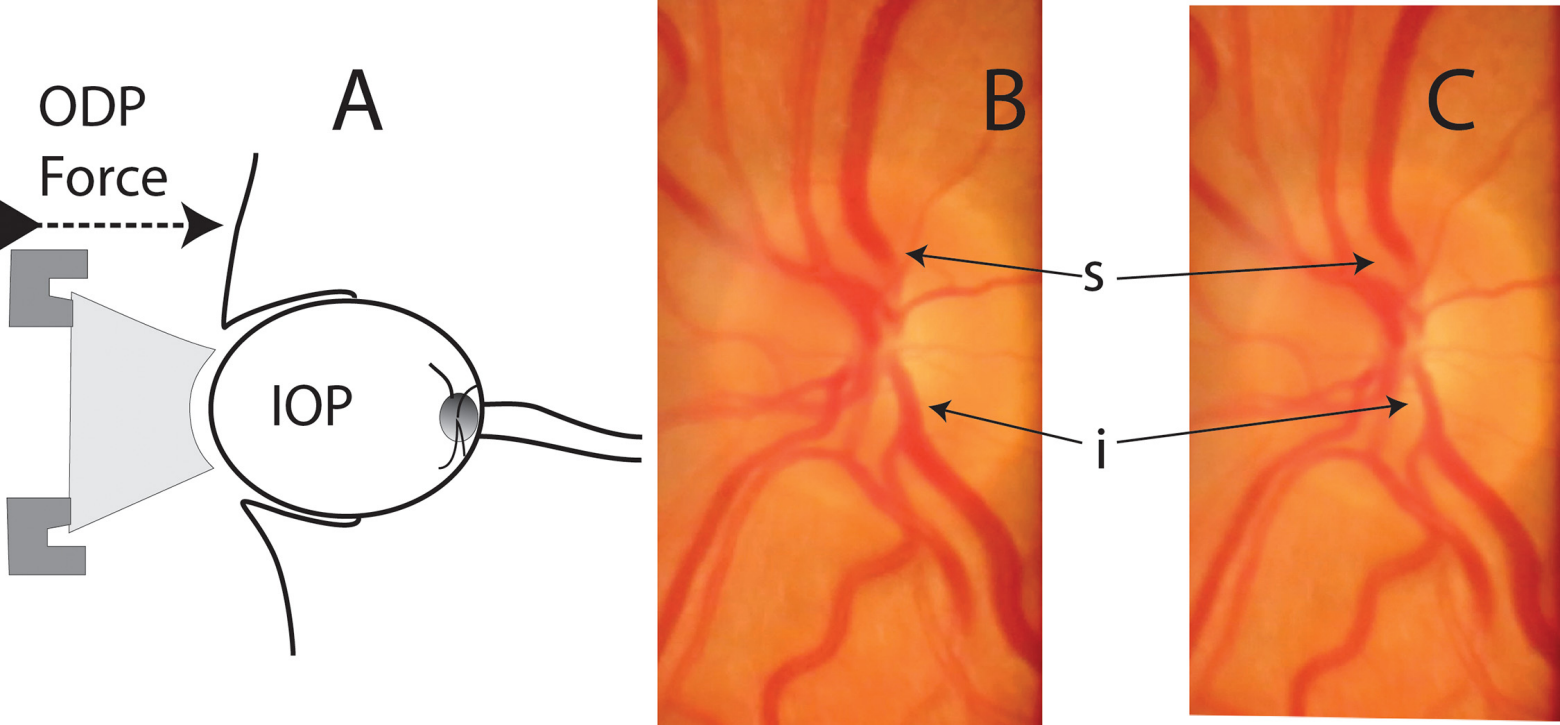
Schematic diagram (A) of ophthalmodynamometric pressure (ODP) being applied through an ophthalmodynamometer to the eye with baseline, post-dilation IOP (IOP). The venous pulsation pressure is the sum of the baseline IOP and ODP. Superior (s) and inferior (i) hemi-veins are pulsating with ‘B’ showing them at dilation and ‘C’ during collapse phase of pulsation.

At the initial visit all patients had a pre-dilation intraocular pressure measurement. If this measurement was greater than 30 mmHg then the subject was given a combination of Timolol 0.5% and Brimonidine 0.2% topically. Gonioscopy was performed to confirm open angle status. The patients were dilated for continued examination once their intraocular pressure fell below 30 mmHg. Following dilation, IOP was remeasured just prior to ophthalmodynamometry and this post-dilation IOP measurement used in the VPP calculation. The IOP measurement prior to dilation was used in the IOP change calculation. The optic discs were examined and the presence or absence of spontaneous venous pulsation in the superior and inferior hemi-veins noted. If pulsation was spontaneous then this meant that VPP was less than or equal to post-dilation IOP by an unknown negative or zero ODP. However we could not measure a negative ODP and so VPP was recorded as being equal to IOP and an ODP score of zero was also recorded. It is worth noting that this censored the data to the left by an unknown negative ODP. When spontaneous venous pulsation was absent in either hemi-vein, the ophthalmodynamometer (Ocudyne, Fig 1)[6] was used to measure the minimum additional pressure (ODP) required to induce pulsation in the superior and inferior hemi-veins. VPP was calculated by adding ODP to the post-dilation IOP.[6] Three measurements of each parameter were recorded and the average value used in analysis. At the final visit, all subjects had IOP measured pre and post-dilation as above. Ophthalmodynamometry was also performed as above.

Visual field hemifield mean deviation was calculated by averaging the pointwise total deviations from the Humphrey visual field printout from each upper and lower hemifield. In order to reduce the effect of measurement variation the IOP measure taken at the invitation visit within six weeks of the initial visit and the initial visit IOP were averaged to give a mean initial IOP. Similarly, the IOP measure taken at the second invitation visit and the final visit IOP were averaged to give a mean final IOP. This was not done in two subjects with initial intraocular pressures greater than 35 mmHg. The change in IOP was calculated as a final mean IOP minus initial mean IOP. Change in hemi-vein VPP was calculated as final hemi-vein VPP minus initial hemi-vein VPP. All changes in treatment were noted during this period and grouped as no change or change, with the changes being beta blocker, prostaglandins or surgery. Carbonic anhydrase inhibitors and alpha-2 agonists were not added to any patient for more than four weeks in this study and so these agents were not included in the treatment analysis. Surgery was trabeculectomy with an antimitotic.

## Statistics

**Fig 2:**
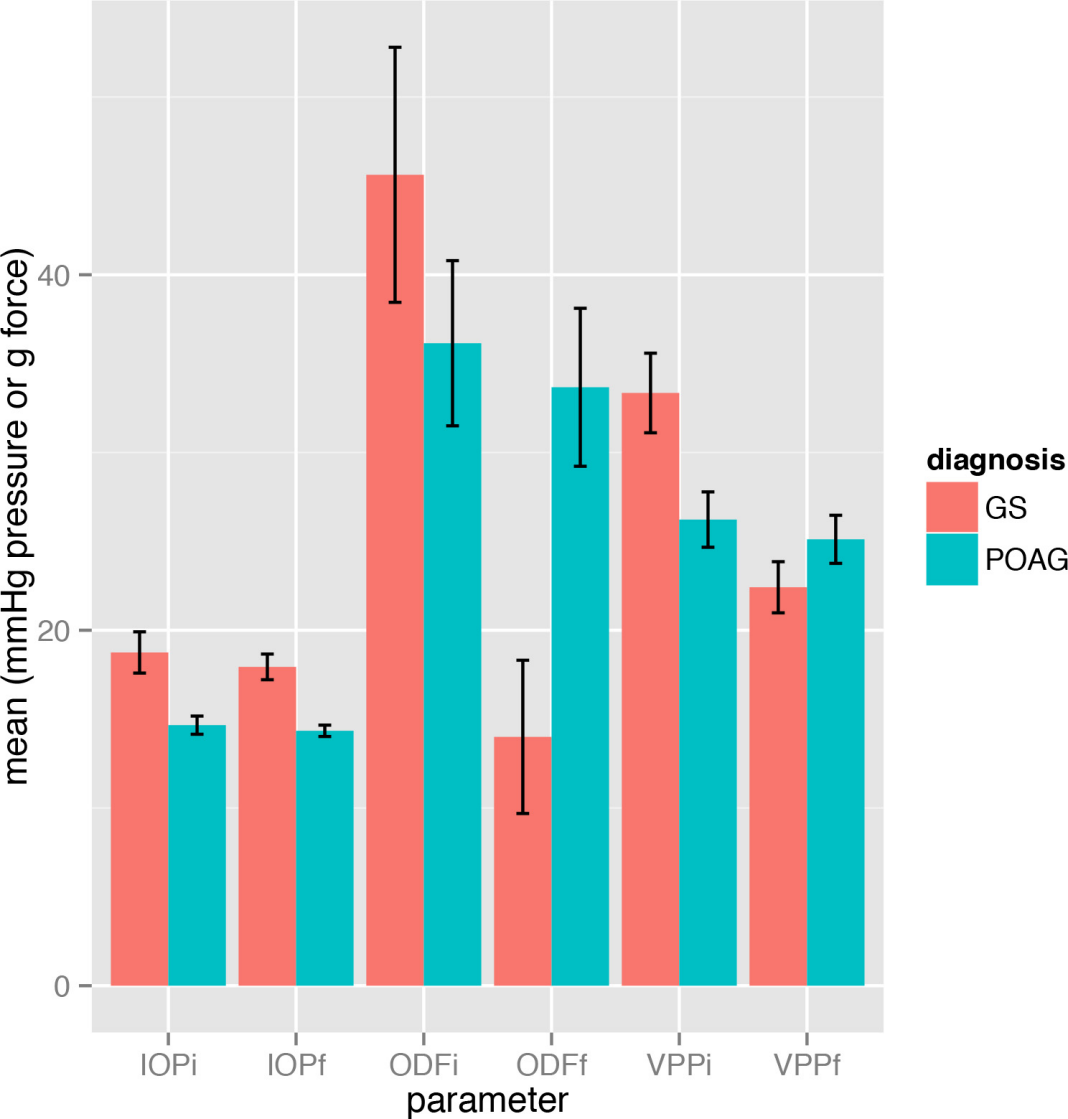
Bar graph showing the means and standard errors for the key parameters: IOP–intraocular pressure (mmHg), ODF–ophthalmodynamometric force (g) and VPP–vein pulsation pressure (mmHg), at the initial (i) and final (f) visits. GS–glaucoma suspect. POAG–primary open angle glaucoma.

All data is expressed as the mean with standard deviation and analysed using R.[18] Data from both upper and lower hemi-veins from each eye was analysed. To account for the multiple measurements from each eye (upper and lower veins) and individuals (right and left), the data was modelled using eye (upper or lower hemi-vein) specific random effects nested within patient (right or left eye) specific random effects.[19] The data was found to have a predominance of zero values for ODP and changes in VPP (Fig 2) due to the fact that negative ODP values at spontaneous venous pulsation cannot be measured and are scored as zero. Due to this unknown censoring and non-normality, a binary approach was used in which reduced VPP was scored as 1 and an increase or no change in VPP was scored as 0. A mixed logistic regression model incorporating the random effects on the scale of the linear predictor was used with reduced hemi-vein VPP as the response variable. The covariates tested were change in IOP, central corneal thickness, hemi-field mean sensitivity loss, time interval between examination, treatment change, sex and age. The superior hemi-field and inferior hemi-vein measurements were compared and vice versa, the inferior hemifield and superior hemi-vein measurements compared.

## Results

Data from 60 eyes of 31 subjects (20 female, 11 male) were analysed (Table 1). The mean age was 60 years (sd 11) with mean time interval between measurements of 13.0 (sd 6.0) months. Of the 60 eyes, 16 were glaucoma suspects and 44 had primary open angle glaucoma. Thirty six eyes had no treatment changes whereas 15 had added prostaglandins, 5 prostaglandins and beta blockers added, 5 trabeculectomy surgery and 3 added beta blockers. Mean change in IOP was -3.4mmHg (sd 8.3) with range from 37mmHg reduction to 5mmHg increase. Mean central corneal thickness was 538μm (sd 40), mean initial intraocular pressure was 18.7 mmHg (sd 9.2). Mean visual field deviation was -5.3 dB (sd 5.8). The mean initial hemi-vein ODP was 12 mmHg (sd 14) and mean change in ODP was -3 mmHg (sd 11). The mean initial hemi-vein VPP was 28 mmHg (sd 14, range 10 to 75mmHg, upper quartile 36mmHg) with mean change in VPP, -4 mmHg (sd 12) reduction. The glaucoma patients had mean initial IOP 14.6mmHg (Fig 2, sd 4.8), initial VPP 26.2mmHg (sd 14.6) and mean hemifield defect -6.7dB (sd 7.4). The glaucoma suspect patients had mean initial IOP 18.7mmHg (Fig 2, sd 6.6), initial VPP 33.3mmHg (sd 12.6) and mean hemifield defect -0.8dB (sd 1.6). Hemi-vein pulsation was spontaneous in 41% of glaucoma patients compared to 28% in suspects (p = 0.28, Chi square test).

**Table 1:** Summary of key subject parameters, with All subject eyes analysed, and data from glaucoma (POAG) eyes and glaucoma suspect (GS) eyes analysed. iIOP and iVPP are initial IOP and VPP respectively. ΔIOP and ΔVPP are change in IOP and VPP respectively. MD is the mean deviation of the sensitivity losses in the upper and lower halves of the visual field. CCT is central corneal thickness.

	number		Age	iIOP	ΔIOP	CCT	MD	iVPP	ΔVPP
All	60	mean	60	18.7	-3.4	538	-5.3	28	-4
		sd	11	9.2	8.3	40	5.8	14	12
POAG	44	mean	60	14.6	-1.3	533	-6.7	26.2	-1
		sd	11	4.8	5.9	42	7.4	14.6	12
GS	16	mean	59	18.7	-9.3	546	-0.8	33.3	-11
		sd	13	6.6	10.8	35	1.6	12.6	11

**Fig 3:**
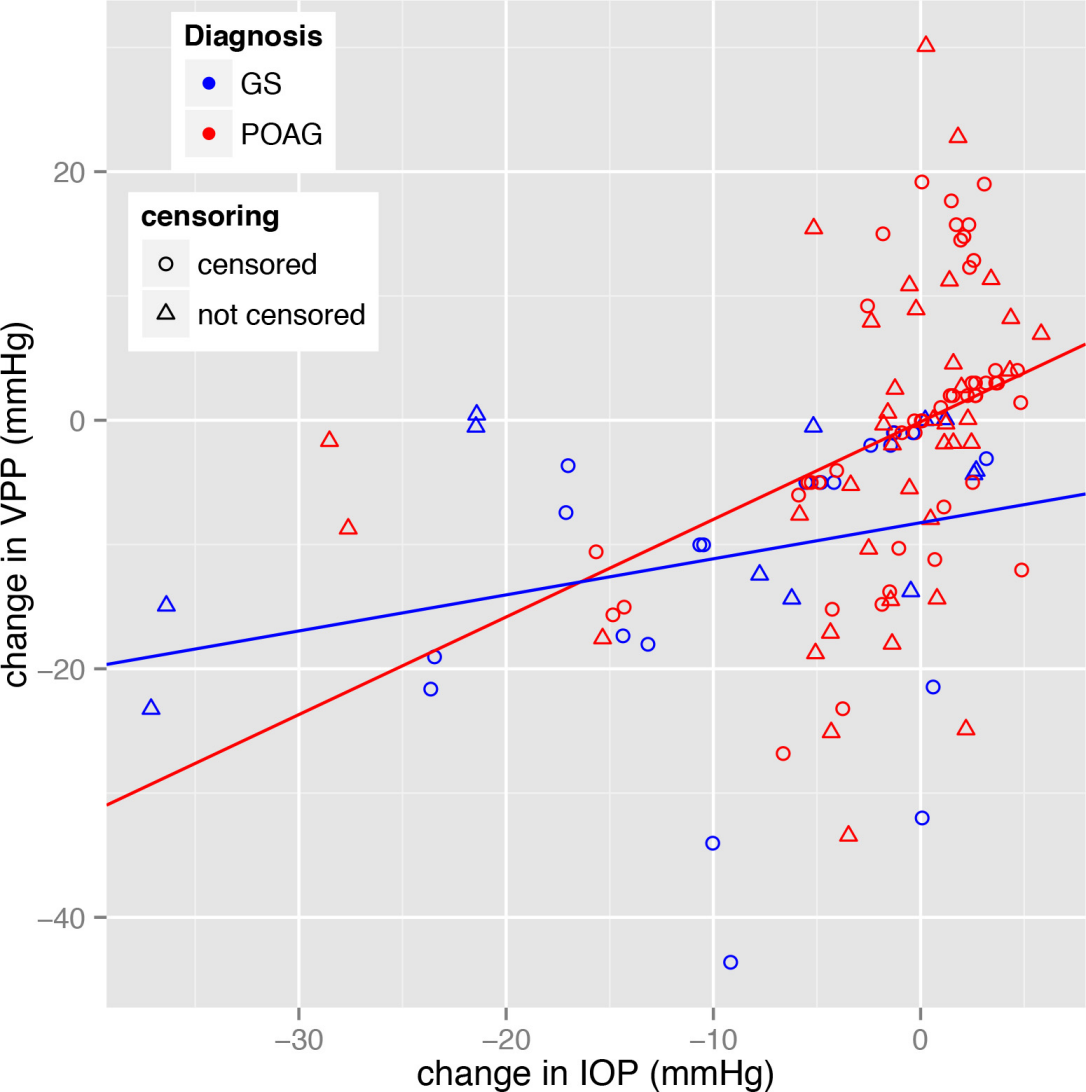
Plot showing change in vein pulsation pressure (VPP) versus change in intraocular pressure (IOP). Observations from upper and lower hemi-veins in both right and left eyes are included. If venous pulsation was spontaneous at initial or final visit, ODF was censored and recorded as zero. Each data point relating to change in VPP that is calculated using a censored ODF value is plotted using a circle (Ο), while a change in VPP calculated from 2 uncensored values is plotted using a triangle (Δ). Glaucoma suspect (GS) eye data is blue and glaucoma (POAG) eye data is red. Lines of best fit for each group are shown.

A strong interaction (p = 0.0069) between change in IOP and diagnosis was found modelling various parameters against reduced VPP. Change in IOP was not associated with reduced VPP when the eye had glaucoma suspect status (p = 0.6228), but was when the eye had glaucoma (p = 0.0005). Increasing age was also associated with a greater likelihood of a reduction in VPP (p = 0.0328, odds ratio 0.94) in glaucoma eyes. None of the other covariates were associated with reduced VPP (minimum p = 0.2007 for central corneal thickness). Using only the glaucoma eye data, the model estimated coefficient was 0.47 resulting in the odds ratio of a reduced VPP being 1.60 per mmHg IOP reduction (95% confidence intervals 1.22 to 2.08). Treatment was broken down into the types of treatment changes and no relationship could be found for any particular treatment type (minimum p = 0.794). Fig 3 shows the change in VPP compared to change in IOP from upper and lower hemi-veins in both eyes.

We recorded IOP at all intervening visits and examined that data. The average time interval between IOP measures was 3.1 (sd 0.8) months. We assumed that an IOP change of 3mmHg or more between initial and final visits was clinically significant, selected those datasets and calculated the time interval from initial visit to the visit when IOP altered by at least 66% of the total change in IOP. We thought this would give an impression of how the IOP changed over the total period. Twenty three eyes had a significant reduction in IOP with mean time to 66% reduction being 2.2 (sd 1.7) months. Nine eyes had a significant rise in IOP with mean time to 66% rise being 8.7 (sd 4.0) months, which was significantly longer that the IOP reduction group (p < 0.0001, Student’s t-test).

## Conclusion

In our glaucoma patients, change in intraocular pressure was strongly associated with change in hemi-vein VPP such that a reduction in intraocular pressure was associated with hemi-vein VPP (p = 0.0005). The major determinants of VPP identified in modelling experiments are cerebrospinal fluid pressure (CSFP) and retinal venous resistance.[7] One quarter of our subjects eyes had initial VPP above 36mmHg but no cases of papilledema were seen. This would be expected if elevated cerebrospinal fluid pressure were the only cause of raised VPP.[20, 21] An additional finding was that lower age was associated with greater likelihood of a reduction in VPP in the glaucoma group (p = 0.0328). We did look to see if age interacted with treatment change (p = 0.1390) or change in IOP (p = 0.9949) in its association with a VPP reduction but could not find any such interaction. Unfortunately, this relatively small dataset does not allow us to further explore the effects of age upon venous properties in a glaucoma population.

It is likely that venous resistance is a significant cause of VPP alteration seen in these subjects. It is possible that the change in VPP is due to a change in retinal vein properties, which may be via venous wall alteration or altered extrinsic compression. The central retinal vein endothelial cells in the lamina cribrosa region are unusually spindle shaped in the normal situation and appear to be exposed to high shear stress, probably from the translaminar pressure gradient.[22] Lowering IOP may reduce this gradient and have a favourable effect upon the venous endothelium. It may also have a direct effect upon lamina cribrosa morphology reducing extrinsic compression.[23–25] It is possible that this postulated venous plasticity is more prominent in younger patients.

The limitations of this study include the fact that relatively few numbers of subjects were suitable for inclusion (n = 32) which limits the statistical power. This does limit the sub-group analysis, particularly our ability to look at the effects of different treatment changes upon the venous pulsation properties. An additional problem was the inability to measure negative values of ODP when venous pulsation was spontaneous. This limited the analytical choices and introduced a possible bias by putting the zero change values into the binary 0 data. We did check for possible bias by including zero change values in the binary 1 value using the same logistic regression model and found a similar significant relationship between change in VPP and change in IOP (p = 0.0001). This study was performed upon glaucoma patients and so the association between change in IOP and change in VPP may be limited to glaucoma patients and not generalizable to other subjects. The fact that IOP reduction occurred so early in the follow-up time course and that we only performed ophthalmodynamometry at the initial and final visits makes it impossible to conclude whether the venous pulsation properties changed early or later. We are unsure why the change in IOP was associated with change in VPP in glaucoma but not suspect eyes. We only had 16 glaucoma suspect eyes and so remain circumspect about drawing conclusions from this relatively small group.

We show that IOP reduction is associated with reduced VPP in glaucoma patients. This suggests that the retinal veins can recover towards more normal pulsation characteristics, implying that a possible reduction in retinal venous resistance is occurring. Whilst we don’t understand the nature or time course of these retinal venous changes, they may indicate possible benefits for glaucoma patients. Additionally, these changes may indicate increased perfusion pressure to the retina and optic nerve over and above that caused by just the IOP reduction.
